# A Volume of Smoke and Very Little Fire

**Published:** 1920-10-16

**Authors:** 


### THE GREAT ILLUSION.

### A Volume of Smoke and very Little Fire.

A WIDELY held opinion—one from which I venture to dissent—is that a great deal is being accomplished to raise the status and extend the influence of women, especially by the Legislature. The most recent Representation of the People Act conferred the parliamentary vote on some six millions of women. A short Act, passed at the end of last year, removed all disabilities in the way of women holding public offices, and now any day we may have a woman Lord Chancellor, Secretary of State for War, or Lord Chief Justice. The same Act, by the way, made it mandatory on women, when called upon, to serve as jurors in courts of law. Then, to come to our own farmyard, there are to be three State Registers for the Trained Nurses of the Kingdom.

All this, and much more in addition.

And yet I have the hardihood to dissent. I see a great volume of smoke and very little fire. Women's votes will doubtless help the nation to progress, and there are frequently cases tried in court where one or two women on the jury will be a godsend. But I fail to see that because of these things the social, economic, or spiritual welfare of women is undergoing any radical change. Does anybody suppose that a State Register can add one cubit to the stature of a trained nurse? It is a striking circumstance, now that Registration is a *fait accompli*, that all the pent-up enthusiasm which underlay the long-drawn-out agitation has evaporated. The unreality of the hopes that were cherished has become manifest, and a calm retrospect shows that the trained nurses of the kingdom have been the witnesses of a great illusion. We shall no doubt have well-filled Registers, but what else beside? Matrons will continue to complain, with something like despair, that the standard of probationers is falling, and that the profession is on the down-grade.

### TRUTHS TO BE REALISED.

It is, I hold, extremely important that the truth should be realised not alone by nurses themselves and hospital authorities, but by the general public. Even the educated public, including members of both Houses of Parliament, are perfectly happy with regard to nurses, honestly believing that they have delivered them out of a horrible pit, and established them upon a rock. If you inquire you will be told that the new legislation will probably provide nurses with good salaries, rapid promotion, and adequate pensions, and if not exactly with these, at least with their equivalent. Nurses have been paid, if not in money, in kind. It is really wonderful how much can be accomplished by newspaper paragraphs and startling headings. "Women forging ahead!" "Six millions of women emancipated!" "Nurses' Registration at last!" And so forth.

I am discussing propaganda, and I ask the reader to tell me how there can be any fruitful propaganda on behalf of the nurse when the vast majority of the British people believe that with much difficulty, and with great credit to themselves, they have brought her salvation? They could not otherwise believe. This was dinned into their ears *ad nauseam*. For full twenty years there was carried on a vigorous campaign of false and pernicious propaganda. Trumpets were blown and drums beaten. Deputation on deputation waited on public men and women. They hung round the corridors of newspaper offices. "They called so loud that all the hollow deep of hall resounded."

For all this, none, of course, will dream of attaching blame to the nurse, for, although she participated in the campaign, she was without guile. She was taught to believe, and she believed, that Registration would confer upon her a monopoly, and that people who nursed for gain, if unregistered, would be liable to punishment. This was a part of the illusion. So far is this from being true that even the Minister of Health shows no inclination to allow her to participate in the reconstruction of our national health. The future health visitor is to be an untrained nurse

### GOOD OUT OF EVIL?

It may possibly happen that from out of evil good will emanate. State Registers will enable British nurses to discover each other by enrolment, and at the same time to realise that they are united by a common bond. It is not too late to begin to pursue after the substance, although so many years and so much vital energy have been wasted in following the shadow. They must, however, be on their guard, because these State Registers will be public property, and, as of old, many mountebanks will be prowling round ready to provide new illusions for the unwary. With great deliberateness the nurse will need to formulate her programme of progress. I am convinced that if she fails to arrive the fault will be her own. The people of this kingdom will, without hesitation, grant the nurse a living wage, a reasonable outlook, and an adequate pension on retirement. The Army was never remarkable for liberality of reward. Hence the pay and status of Army nurses may be taken as a modest standard. British people will never deny this to the trained women who devote their lives to the nursing of the nation's sick poor.

But the demand must be presented. IERNE.

